# Immunisation of healthcare workers in the Nordic countries: Variation in recommendations and practices and a lack of assessment

**DOI:** 10.2807/1560-7917.ES.2021.26.4.1900555

**Published:** 2021-01-28

**Authors:** Timothee Dub, Bolette Søborg, Peter Henrik Andersen, Thorolfur Gudnason, Hanne Nøkleby, Ann Lindstrand, Rose-Marie Carlsson, Hanna Nohynek

**Affiliations:** 1 Department of Health Security, Finnish Institute for Health and Welfare (THL), Helsinki, Finland; 2 European Programme for Intervention Epidemiology Training (EPIET), European Centre for Disease Prevention and Control (ECDC), Stockholm, Sweden; 3 Danish Health Authority, Copenhagen, Denmark; 4 Department of Infectious Disease Epidemiology and Prevention, Statens Serum Institut, Copenhagen, Denmark; 5 Centre for Health Security and Communicable Disease Control, Directorate of Health, Reykjavik, Iceland; 6 Norwegian Institute of Public Health, Oslo, Norway; 7 Unit for Vaccination Programmes, Public Health Agency of Sweden, Solna, Sweden

**Keywords:** vaccine preventable diseases, healthcare workers, Nordic countries

## Abstract

Healthcare workers (HCWs) are at increased risk of both exposure and transmission of infectious disease. Two European Union (EU) directives state that health services are responsible for assessing their employees’ potential exposure to infectious diseases and offering immunisation free of charge. We assessed current policy for immunisation of HCWs and the availability of vaccine coverage data in the Nordic countries by surveying national vaccination experts in Denmark, Finland, Iceland, Norway and Sweden, as well as Swedish county medical officers (CMOs). All national experts and 17 of 21 Swedish CMOs responded. All EU countries had transposed the European directives into national law, while Norway and Iceland had similar national legislation. Recommendations or guidelines were issued in Denmark, Finland, Iceland, Norway and 15 of 17 responding Swedish counties. The range of diseases covered differed by countries and Swedish counties. HCW vaccine coverage data were not systematically collected; incomplete estimates were only available for Finland and two Swedish counties. In conclusion, recommendations or guidelines exist in the Nordic countries, but their impact cannot be assessed, as vaccine uptake among HCWs is not currently measured. Systematic collection of data is a necessary step towards improving HCW immunisation policy and practice in the Nordic countries.

## Background

Healthcare workers (HCWs) are at risk of exposure to and transmission of infectious diseases, including a number of vaccine-preventable diseases (VPDs), because of frequent contact with either contagious or vulnerable patients. Transmission of hepatitis B to a HCW following percutaneous injury has been found to vary from 6–30% [1], while a meta-analysis concluded that influenza incidence among HCWs—both vaccinated and unvaccinated—was higher than among healthy adults in the general population, with incidence rate ratios of 5.4 and 3.4, respectively [2]. Both the World Health Organization [3] and the United States Center for Disease Control and Prevention [4] recommend immunisation of HCWs against an extensive list of pathogens—including measles, hepatitis B, pertussis and influenza—depending on their individual risk of exposure.

In 2018, European Parliament called upon European Union (EU) countries to ensure that all HCWs are sufficiently vaccinated and upon the Commission to address HCW vaccination rates [5]. Also in 2018, the European Council issued a recommendation to strengthen cooperation against VPDs, mentioning that countries should address HCW vaccination coverage rates if they do not meet the national recommendations to protect HCWs and their patients [6]. Two directives concerning worker protection that are applicable to HCWs were issued at the European level. The EU Directive 2000/54/EC, on the protection of workers from risks related to exposure to biological agents at work, states that if any activity of an employee is likely to involve a risk of exposure to biological agents, the employer is responsible for assessing potential risk and implementing all necessary protection measures, including providing immunisation free of charge to personnel when relevant [7]. The European Agency for Safety and Health at Work (EU-OSHA) Directive 2010/32/EU, on prevention from sharp injuries in the hospital and healthcare sector, also states in clause 6 (Elimination, prevention and protection) that if there is a risk to the safety and health of workers due to their exposure to biological agents for which effective vaccines exist, all workers—including students delivering healthcare—should be offered vaccination free of charge [8]. Employee obligations are limited to (i) immediately reporting potential incidents to the employer or their representative, as per Directive 2000/54/EC, and (ii) taking care of their own safety and health—as well as that of other persons affected by their actions at work—in accordance with their training and the instructions given by their employer, as per Directive 2010/32/EU.

In 2017 and 2018, the role of HCWs in the transmission of VPD in Europe was demonstrated in several measles outbreaks, including in Norway and Sweden, countries where measles-mumps-rubella (MMR) immunisation coverage is high [9]; hence, the European Centre for Disease Prevention and Control recommends that countries ensure that all HCWs are immunised against measles [10]. Similar advice concerning seasonal influenza was also given in an effort to decrease the risk of HCWs transmitting infections to their vulnerable patients [11].

In March 2018, Finland introduced a new section in the infectious diseases act that makes employers responsible for ensuring that social care workers and HCWs working with vulnerable patients are protected, which was defined as either already immune to or vaccinated against measles and varicella, and vaccinated against pertussis and seasonal influenza [12]. As this new mandate elicited mixed reactions among HCWs and employers, we wanted to find out how HCW immunisation recommendations were organised in the Nordic countries, i.e. Denmark, Norway, Iceland and Sweden, compared with Finland.

Our objective was to assess Nordic countries’ HCW immunisation recommendations or guidelines, laws and practices—along with vaccine coverage data, if available—in order to provide Finnish and other Nordic countries’ experts and stakeholders with comparable information on how to improve current national recommendations or guidelines and their implementation.

## Survey on recommendations or guidelines to immunise healthcare workers

We conducted a web-based survey among national public health experts, members of National Immunisation Technical Advisory Groups involved in issuing recommendations on HCW immunisation in Nordic countries and national experts, as well as Swedish county medical officers (CMOs) (n = 21), because of the decentralised organisation of healthcare in Sweden. The survey was conducted from April to June 2018 using Webropol 2.0 and was followed by phone discussions or email exchanges around points requiring clarification. We asked for information on recommendations or guidelines for eight defined diseases (hepatitis A, hepatitis B, influenza, measles, mumps, pertussis, rubella and varicella), as well as ‘other recommendations’. The survey sent to national experts contained 55 questions, including 33 closed-ended questions, while the one sent to Swedish CMOs contained 39 questions, including 23 closed-ended questions. Respondents were also given the possibility to share any official documents and references regarding HCW immunisation regulations, if available. National experts from all Nordic countries (one expert per country, except for Denmark, for which two experts were surveyed) and 17 of the 21 Swedish CMOs responded to the survey.

HCWs were defined as all persons involved in patient care, such as healthcare professionals, residents, students, laboratory staff, and administrative and service personnel, as well as individuals involved in public health such as field workers, epidemiologists, laboratory staff and community health workers.

Individuals were defined as protected against an infectious disease if they had contracted the disease and developed long-lasting immunity proven through documentation of immunity, such as adequate IgG levels, or had been correctly immunised against a disease following the vaccination programme of the country, including regular boosters if required.

Recommendations were defined as protocols that should be followed by HCWs’ employers in order to ensure their protection, while guidelines were defined as advice for HCWs’ employers to consider.

## Ethical statement

All public health professionals participated in this survey and discussion on a voluntary basis and no personal data were collected; hence, ethical committee review and written informed consent were not required.

## Transposition of European directives into national laws

EU Directive 2000/54/EC, on the protection of workers from risks related to exposure to biological agents at work [7], the EU-OSHA Directive on prevention from sharp injuries in the hospital and healthcare sector [8] and Directive 2000/54/EC on biological agents at work, had been transposed into national laws or regulations in the EU countries Denmark [13,14], Finland [15,16] and Sweden [17], while Norway [18] and Iceland [19] had similar legislation.

### National recommendations or guidelines

Disease-specific immunisation recommendations or guidelines for HCWs associated with legislation existed at the national level in Finland [20], Iceland [21] and Norway [22], while in Denmark, national guidelines issued by the Statens Serum Institut were associated with legislation issued by the Authority for Work Environment in Denmark [14].

The scope of the recommendations, the categories of HCWs covered and the number of VPDs covered differed between countries, ranging from three VPD guidelines in Denmark (i.e. seasonal influenza for all HCWs, hepatitis B for HCWs with a potential risk of cutting or stabbing injuries [23] and pertussis immunisation for personnel in contact with infants and newborns) to 10 recommendations for all HCWs in Iceland (i.e. diphtheria, hepatitis B, influenza, measles, mumps, pertussis, pneumococcal disease, poliomyelitis, rubella and tetanus) (Table) [21].

**Table:** Summary of national recommendations or guidelines for healthcare worker immunisation, by country and vaccine-preventable disease, Nordic countries, 2018

VPDs	Denmark	Finland	Iceland	Norway
Diphtheria	None	None	All HCWs	All HCWs
Hepatitis B	HCWs in long-term care facilities, maternity wards or with a significant risk of transmission of infection and sting lesions	All HCWs	All HCWs	All HCWs
Influenza	All HCWs	All HCWs	All HCWs	All HCWs
Measles	None	All HCWs	All HCWs	All HCWs
Mumps	None	None	All HCWs	None
Pertussis	HCWs treating infants	HCWs treating infants	All HCWs	All HCWs
Pneumococcal disease	None	None	All HCWs	None
Poliomyelitis	None	None	All HCWs	All HCWs
Rubella	None	None	All HCWs	All HCWs
Tetanus	None	None	All HCWs	None
Tuberculosis	None	None	None	HCWs working with at-risk patients
Varicella	None	All HCWs	None	All HCWs

HCWs: healthcare workers; VPD: vaccine-preventable disease.

Indicated guidelines and recommendations are according to each country’s national HCW protection Directives.

In comparison, in Finland five VPDs were covered by recommendations. Protection against measles, varicella, influenza and hepatitis B was required for all HCWs, while pertussis immunisation was only recommended for personnel caring for infants [20]. These recommendations were broader than those issued by the Finnish Institute of Occupational Health in 2007 [24], in order to take into consideration recent epidemiological changes, including the re-emergence of measles.

In Norway, pertussis, poliomyelitis, rubella, measles, varicella, diphtheria, hepatitis B and influenza (since season 2018/19) immunisations were recommended for all HCWs. Tuberculosis immunisation was additionally recommended to HCWs working with at-risk patients. Hence, a total of nine VPDs were covered by the Norwegian recommendations.

### Regional recommendations in Sweden

Of the 17 Swedish CMOs who answered our survey, 15 responded that immunisation recommendations were issued at the regional level. The number of diseases covered ranged from two in the Dalarna region (i.e. hepatitis B and influenza) to nine in the Gävleborg and Jämtland regions (Supplementary Table S1). Only six CMOs were aware that the recommendations issued were associated with legislation in the provisions on contagious risks from the Swedish Work Environment Authority [17] and in the statutes from the National Board of Health and Welfare about systematic quality work [25].

### Obligations because of legislation associated with recommendations

In theory, according to the legislation associated with the recommendations, in Norway [18] and Denmark it was the employers’ responsibility to assess HCWs’ risk of infection, offer immunisation and cover related costs. In Iceland, the employer was expected to ensure that personnel were fully vaccinated upon employment, offer immunisation if necessary and re-assess HCWs’ immunisation status every 10 years [21]. In Finland, legislation was less precise and only mentioned that it was the responsibility of the employer to ensure that personnel were protected against the aforementioned infectious diseases when taking care of vulnerable patients [20].

In practice, in Iceland the employers were responsible for organising regular immunisation campaigns and controlling immunisation of HCWs upon recruitment; in Norway, Denmark and Finland, the employers were responsible for assessing HCWs’ risk of infection and providing immunisation accordingly, which was in complete agreement with the spirit of the EU-OSHA Directives 2010/32/EU [8] and 2000/54/EC [7]. ]. In Finland, the employer had to assess a HCW’s immunisation status upon recruitment—but was not permitted to ask for written proof [25]—and to organise HCWs’ access to immunisation through occupational medicine, while in Norway employers were only responsible for the organisation of HCWs’ access to immunisation through occupational medicine.

Iceland was the only country where the recommendations targeted both employers (health services) and their employees (HCWs). Indeed, HCWs had to provide documented proof of immunisation upon employment and comply with regular occupational health visits.

None of the recommendations or guidelines were associated with mandatory immunisation policies.

## Assessment of HCW vaccine coverage

No systematic collection of vaccination uptake data for HCWs was performed in any country. However, in Finland, a survey of specialised care wards showed that average influenza immunisation coverage of nursing staff was 84% during the 2017/18 season. Two Swedish CMOs had incomplete data regarding vaccine coverage. In Sweden’s Jämtland region, a survey on measles immunisation in 3,718 HCWs showed that only 74% (2,738) were properly protected, while in the Skåne region it was shown that following the implementation of the recommendation on influenza immunisation of HCWs, coverage rose from 22% to 37% (data not shown).

## Conclusions

Immunisation of HCWs is not mandatory in the Nordic countries, even though in Iceland HCWs have to provide documented proof of immunisation before employment and must comply with regular occupational health visits. Recommendations or guidelines for voluntary protection of HCWs against VPDs were issued in all other Nordic countries. In Denmark, Sweden and Finland (EU countries), the EU-OSHA Directives regarding prevention of occupational infectious exposures [7,8] were transposed into national laws or regulations, but their application into practice differed between countries. Denmark provided national guidelines to employers through the Statens Serum Institut, while recommendations in Sweden were issued at the regional level. There were also differences between countries regarding the categories of HCWs targeted by the recommendations and the spectrum of VPDs. This heterogeneity was already evidenced at the European level in a report published in 2012, when various recommendations were issued in 24 countries, including 10 countries where—depending on the VPD—recommendation was mandatory [26].

As no systematic monitoring of immunisation uptake or measurement of effectiveness of HCW vaccination programmes was carried out, we could not establish whether recommendations or guidelines were properly implemented and followed, nor whether they would lead to increased coverage, thereby decreasing the risk of HCW involvement in VPD transmission in healthcare settings. Developing an information system or immunisation register of HCWs could be a valuable tool to monitor coverage. However, the EU General Data Protection Regulation, passed in 2016 and applied in full in 2018, would not allow employers to collect and have access to this information regarding their personnel [27].

Our main recommendation regarding potential improvement of HCW immunisation guidance would be to develop a system to monitor vaccination coverage among different professional groups, which could be done in collaboration with HCW unions or associations, occupational health providers, employers (hospitals or other healthcare providers) and/or national health institutes or authorities. This would make it possible to conduct a proper assessment of the impact of the existing recommendations and/or guidelines, such as whether they improve HCW safety, prevent transmission from HCWs to at-risk patients and decrease the risk of HCW-mediated outbreaks.

## Supplementary Data

524000 SupplementaryTable
