# Supplementary material for: Immunisation of healthcare workers in the Nordic countries: Variation in recommendations and practices and a lack of assessment
Source: Euro Surveill. 2021 Jan 28;26(4):1900555. doi: 10.2807/1560-7917.ES.2021.26.4.1900555 (PMC7848784; doi:10.2807/1560-7917.ES.2021.26.4.1900555)
Supplement: SupplementaryTable [file 1900555_Supplement.pdf]

This supplementary material is hosted by *Eurosurveillance* as supporting information alongside the article 'Immunisation of healthcare workers in the Nordic countries: Variation in recommendations and practices and a lack of assessment' on behalf of the authors who remain responsible for the accuracy and appropriateness of the content. The same standards for ethics, copyright, attributions and permissions as for the article apply. Supplements are not edited by *Eurosurveillance* and the journal is not responsible for the maintenance of any links or email addresses provided therein.

SUPPLEMENTARY TABLE S1. Summary of regional recommendations for healthcare workers (HCW) immunisation, according to HCW protection directives: Range of diseases and HCW categories covered in Sweden as of spring 2018

County	Diphteria	Hepatitis A	Hepatitis B	Influenza	Measles	Mumps	Pertussis	Poliomyelitis	Rubella	Tetanus	Tuberculosis	Varicella
Dalarna	None	None	All HCWs	All HCWs	None	None	None	None	None	None	None	None
Kalmar	None	None	None	All HCWs	All HCWs	None	All HCWs	None	None	None	None	None
Gävleborg	All HCWs	None	All HCWs	All HCWs	HCWs caring for pregnant women and children aged < 18 months	HCWs caring for pregnant women and children aged < 18 months	None	All HCWs	HCWs caring for pregnant women and children aged < 18 months	All HCWs	None	All HCWs
Gotland	None	None	All HCWs	All HCWs	All HCWs	None	None	None	None	None	None	None
Jämtland	Depending on HCWs' risk of exposure	None	Depending on HCWs' risk of exposure	All HCWs	Depending on HCWs' risk of exposure	Depending on HCWs' risk of exposure	Depending on HCWs' risk of exposure	None	Depending on HCWs' risk of exposure	None	Depending on HCW's medical history	Depending on HCWs' risk of exposure
Jönköping	None	None	HCWs at risk of blood exposure	All HCWs	All HCWs	None	HCWs with frequent contact with infants	None	All HCWs	None	None	In case of frequent contact with infants and depending on HCWs's risk of exposure
Kronoberg	None	None	None	None	All HCWs	None	None	None	None	None	None	Depending on HCWs' risk of exposure
Norrbotten	None	None	All HCWs	All HCWs	All HCWs	None	None	None	None	None	All HCWs	All HCWs
Skåne	None	None	All HCWs	All HCWs	All HCWs	None	None	None	Female HCWs of childbearing age	None	Depending on HCW's medical history	None
Södermaland	None	All HCWs	All HCWs	All HCWs	All HCWs	All HCWs	All HCWs	None	All HCWs	None	All HCWs	All HCWs
Stockholm	ENT and Anesthesiology HCWs	None	All HCWs	All HCWs	All HCWs	None	None	None	None	None	None	Maternal, childbirth and neonatal care or caring for immunosuppressed patients
Värmland	None	None	All HCWs	All HCWs	All HCWs	None	None	None	None	None	None	None
Västerbotten	None	None	All HCWs	All HCWs	All HCWs	All HCWs	None	None	All HCWs	None	None	Targeted groups of HCWs
Västernorrland	None	None	All HCWs	All HCWs	All HCWs	All HCWs	None	None	All HCWs	None	None	None
Västmanland	None	None	All HCWs	All HCWs	All HCWs	None	None	None	None	None	None	None

HCWs: healthcare workers.
